# Influence of Marginal Tooth Preparation Designs on Periodontal Health and Long-Term Stability: A Narrative Review

**DOI:** 10.3390/jcm14197038

**Published:** 2025-10-04

**Authors:** Tareq Hajaj, Mihai Rominu, George Dumitru Constantin, Maria Dobos, Ioana Veja

**Affiliations:** 1 Department of Prostheses Technology and Dental Materials, Faculty of Dentistry, “Victor Babes” University of Medicine and Pharmacy, 2 Eftimie Murgu Sq., 300041 Timisoara, Romania; tareq.hajaj@umft.ro (T.H.); rominu.mihai@umft.ro (M.R.); 2 Research Center in Dental Medicine Using Conventional and Alternative Technologies, Faculty of Dental Medicine, “Victor Babes” University of Medicine and Pharmacy of Timisoara, 9 Revolutiei 1989 Ave, 300070 Timisoara, Romania; 3 Discipline of Clinical Practical Skills, Department I Nursing, Faculty of Medicine, “Victor Babes” University of Medicine and Pharmacy, 2 Eftimie Murgu Sq., 300041 Timisoara, Romania; 4 Department of Dental Medicine, Faculty of Dentistry, “Vasile Goldiș” Western University of Arad, 310025 Arad, Romania; veja.ioana@uvvg.ro

**Keywords:** tooth preparation, chamfer, shoulder, Biologically Oriented Preparation Technique (BOPT), periodontal health, gingival stability, digital dentistry

## Abstract

**Background**: Tooth preparation design strongly influences the long-term success of fixed prosthodontic restorations, affecting periodontal stability and esthetic outcomes. Conventional horizontal designs such as chamfer and shoulder remain widely used but present biological and technical limitations. The Biologically Oriented Preparation Technique (BOPT), a vertical approach, has been proposed as an alternative. **Methods**: This narrative review synthesizes evidence from clinical trials, histological investigations, and systematic reviews, comparing horizontal preparations with BOPT with emphasis on periodontal parameters and the role of digital workflows. **Results**: Horizontal designs provide predictable outcomes but may predispose to inflammation and marginal instability, especially with subgingival margins. BOPT has been associated with greater gingival thickness, stable probing depths, and favorable esthetic results, with prospective studies reporting stability for up to 10 years. Integration with CAD/CAM workflows appears to enhance precision and reproducibility. **Conclusions**: BOPT shows promising periodontal and esthetic benefits, particularly in thin gingival biotypes and esthetically demanding cases. However, current evidence is limited, and the available studies do not allow firm conclusions about superiority over conventional designs. Further randomized controlled trials with large cohorts and long-term follow-up are required before definitive clinical recommendations can be made.

## 1. Introduction

In fixed prosthodontics, tooth preparation design is a key determinant for the long-term success, esthetic integration, and periodontal stability of restorations. The configuration and position of the finishing line critically determine how the interaction between restorations with gingival and peri-implant soft tissues.

Traditionally, two preparation categories have been described: horizontal preparations, such as chamfer and shoulder, and vertical preparations, including feather-edge techniques [1,2,3,4,5,6,7]. Horizontal finishing lines are predictable and widely taught, but evidence indicates they may predispose to plaque accumulation, inflammation, and marginal recession particularly when extended subgingivally [8,9,10].

To address these limitations, the Biologically Oriented Preparation Technique (BOPT) introduced by Ignacio Loi in 2008 [11] proposed a vertical, edgeless concept. This vertical preparation concept eliminates the pre-existing emergence profile and uses immediate temporization to guide gingival adaptation, promoting increased soft tissue thickness, stable probing depths, and long-term margin stability [12,13,14]. Clinical studies have demonstrated its ability to promote soft tissue thickening, stable probing depths, and long-term margin stability, with prospective trials confirming favorable outcomes after up to 10 years [12,13,14].

Advances in digital dentistry have further enhanced the applicability of vertical preparations. Intraoral scanning, CAD/CAM workflows, and virtual gingiva modeling enable precise reproduction of the emergence profile and more predictable transfer from provisional to definitive restorations [15,16,17,18].

Clinically, the choice between chamfer, shoulder, and BOPT should be individualized: while BOPT offers advantages in thin gingival biotypes and esthetically demanding anterior regions, horizontal designs remain reliable in posterior areas with lower esthetic demands and greater biomechanical requirements [8,12,19].

This review synthesizes clinical, histological, and experimental evidence to critically compare horizontal and vertical preparations, with particular emphasis on BOPT, evaluating their biological, mechanical, and esthetic implications in contemporary prosthodontics.

### Objective

The primary objective of this review is to critically assess and compare the biological, clinical, and esthetic outcomes of conventional horizontal preparations (chamfer and shoulder) with vertical approaches such as the Biologically Oriented Preparation Technique (BOPT). Special emphasis is placed on periodontal health parameters—including probing depth, gingival inflammation, margin stability, and soft tissue thickness—that have been highlighted in prospective clinical trials and systematic reviews as crucial indicators of long-term success [6,17,18].

Another important objective is to evaluate the contribution of analog and digital workflows in shaping the emergence profile of BOPT restorations, with recent studies demonstrating the growing reliability of intraoral scanning and CAD/CAM systems in managing subgingival contours [8,19,20]. The review also considers the mechanical implications of preparation design, including tooth structure reduction [3] and stress distribution in endodontically treated teeth restored with zirconia crowns [7].

Finally, the review explores clinical outcomes of BOPT in implant-supported restorations, particularly with convergent collar implants and customized healing abutments, which have shown favorable effects on peri-implant tissues [21,22,23,24,25,26]. It also addresses the level of professional awareness and adoption of vertical preparation techniques, identifying barriers to broader implementation and underscoring the relevance of the prosthetic adaptation profile as a novel framework for achieving predictable long-term rehabilitation [14,16].

## 2. Materials and Methods

This paper was designed as a narrative review, with elements of structured searching and study selection similar to a scoping review. A formal systematic review was not attempted, as the heterogeneity of study designs and outcomes precluded meta-analysis. This review was conducted as a narrative synthesis of the available evidence on the influence of tooth preparation designs on periodontal health and long-term stability. A comprehensive literature search was performed between September 2023 and January 2025 using four electronic databases: PubMed/MEDLINE, Scopus, Web of Science, and Google Scholar.

The search strategy combined keywords and Boolean operators related to preparation design and periodontal outcomes, including “tooth preparation”, “chamfer”, “shoulder”, “Biologically Oriented Preparation Technique”, “BOPT”, “vertical preparation”, “biologic width”, “periodontal health”, “gingival stability”, and “emergence profile”. Reference lists of systematic reviews and relevant clinical studies were also screened manually to identify additional eligible publications.

Inclusion criteria were as follows: (a) studies published in English between January 2000 and January 2025; (b) clinical trials, prospective or retrospective studies, systematic or narrative reviews, in vitro investigations, and histological studies; and (c) research addressing conventional horizontal preparations (chamfer, shoulder) or vertical preparations (BOPT, feather-edge).

Exclusion criteria included the following: (a) case reports, conference abstracts, and opinion papers without original data; (b) studies unrelated to full-coverage restorations or periodontal outcomes; and (c) implant studies not specifically evaluating preparation design or emergence profile.

After duplicate removal and eligibility screening, 112 articles were included in the final synthesis. Selection was performed independently by two authors, with disagreements resolved through discussion until consensus was achieved. Extracted data were categorized into domains covering periodontal parameters, preparation geometry, analogical versus digital workflows, esthetic outcomes, survival and complication rates, and long-term stability.

Given the heterogeneity of study designs and outcomes, direct quantitative comparison or meta-analysis was not feasible. As this is a narrative review, no formal risk of bias tool was applied; however, study selection and data extraction were performed independently by two authors to enhance transparency.

No formal risk of bias or quality appraisal tool was applied, which is consistent with the scope of a narrative review. However, heterogeneity in study designs and sample sizes was acknowledged and considered in the interpretation of findings.

## 3. Results

A total of 112 studies met the inclusion criteria and were synthesized in this review. The findings are presented according to periodontal, mechanical, esthetic, and digital domains.

### 3.1. Periodontal Parameters

Long-term prospective studies (sample sizes ranging from 40 to 85 patients; follow-up periods of 6–10 years) reported that BOPT maintained stable probing depths (mean variation <0.2 mm) and significantly increased gingival thickness compared with horizontal designs [9,17,18]. One 10-year trial involving 77 restorations confirmed superior periodontal stability and gingival volume preservation in BOPT compared with chamfer and shoulder preparations [17]. By contrast, chamfer and shoulder preparations, though predictable in the short term, have been linked to progressive gingival recession and papilla loss, particularly in anterior esthetic regions [7,8,23].

### 3.2. Gingival Inflammation

Clinical investigations with sample sizes between 30 and 60 patients demonstrated that subgingival chamfer or shoulder margins were associated with higher plaque indices and bleeding on probing (BOP > 25%) after 2–3 years [8,23]. In contrast, BOPT restorations showed significantly lower BOP scores (<10%) and reduced plaque accumulation over comparable follow-up periods [9,17,18].

### 3.3. Tooth Reduction and Biomechanics

In vitro analysis has demonstrated that preparation geometry directly influences the extent of tooth reduction. A recent study by Real-Voltas et al. (2025) showed that BOPT and its modification (BOPTm) preserve more dental structure in anterior teeth compared to chamfer and shoulder, while still ensuring functional retention [3]. Finite element analysis confirmed that marginal design affects stress concentration in both teeth and restorations, with BOPT showing slightly higher marginal stress values but remaining within clinically acceptable limits [7].

### 3.4. Esthetic Outcomes and Patient Satisfaction

Prospective clinical trials (*n* = 45–80 patients; follow-up 6–10 years) reported higher esthetic scores for BOPT restorations compared with horizontal designs, particularly in anterior teeth (mean satisfaction score >90% vs. ~75%) [9,17,18]. One 10-year study further confirmed stable gingival margins and high esthetic predictability, with >95% of patients rating their restorations as satisfactory or highly satisfactory [17]. In contrast, chamfer and shoulder preparations provide initially acceptable esthetics, but satisfaction scores tend to decrease over time as marginal recession develops [23].

### 3.5. Implant-Supported Restorations

Clinical evidence supports the application of BOPT principles in implant prosthodontics. Studies with convergent collar implants and customized healing abutments reported improved peri-implant soft tissue stability and keratinized mucosa width compared with conventional implant protocols [21,25,26].

### 3.6. Digital Workflows

Multiple studies demonstrated the successful integration of digital workflows into vertical preparation protocols. Intraoral scanning and CAD/CAM technologies enable precise reproduction of the emergence profile and facilitate accurate transfer of provisional contours to definitive restorations [8,10,11,19,20]. This improved accuracy in reproducing the emergence profile has been associated with reduced plaque accumulation and lower bleeding on probing scores, as well as more stable probing depths and gingival margins in BOPT cases compared with conventional workflows [8,19,20]. Recent digital protocols also incorporate virtual gingiva modeling and finish line identification, which improve the transfer of provisional contours to definitive restorations. These advances contribute to clinically relevant endpoints such as reduced plaque accumulation, lower bleeding on probing, stable probing depths, and improved margin stability in BOPT cases [11,20].

### 3.7. Summary of Comparative Outcomes

**Figure 1:**
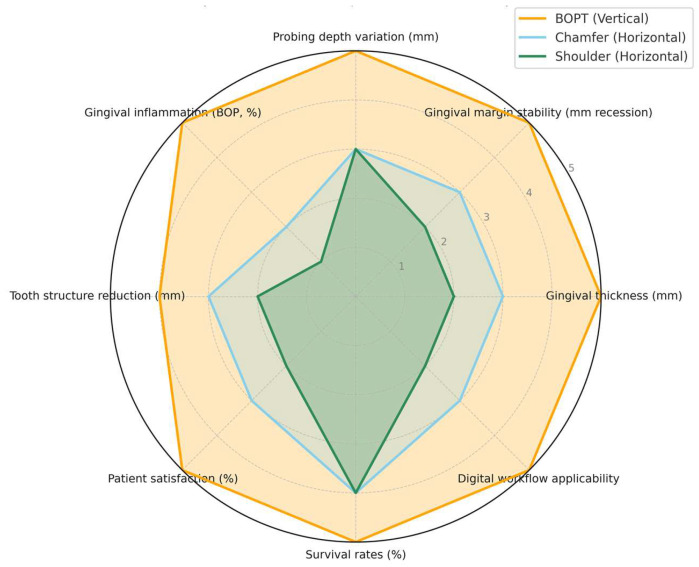
Radar chart comparing clinical outcomes of Biologically Oriented Preparation Technique (BOPT), Chamfer, and Shoulder preparations. Scores were normalized on a 0–5 scale (0 = least favorable, 5 = most favorable) using ranges reported in prospective clinical trials, systematic reviews, and in vitro studies [3,7,8,9,17,18,19,20,21,22,23]. Parameters include gingival thickness (mm change), gingival margin stability (mm recession), probing depth variation (mm), gingival inflammation (BOP, %), tooth structure reduction (mm), patient satisfaction (%), survival rates (% at 5–10 years), and digital workflow applicability. Values represent synthesized evidence from multiple studies, not a single dataset.

The comparative performance of BOPT, chamfer, and shoulder preparations across periodontal, biological, and esthetic parameters is synthesized in Table 1 and illustrated in Figure 1, highlighting the superior stability and clinical predictability of BOPT.

The table summarizes findings from prospective clinical trials, in vitro investigations, and systematic reviews, focusing on gingival thickness, margin stability, probing depth, inflammation, tooth structure reduction, esthetic outcomes, survival rates, and digital workflow integration [3,7,8,9,12,13,19,20,21,22].

The figure compares eight parameters: gingival thickness, margin stability, probing depth, gingival inflammation/bleeding on probing, tooth reduction, esthetic outcomes, survival rates, and digital workflow applicability. Data were synthesized from key clinical and laboratory studies [3,7,8,9,10,17,18,19,20,23]. BOPT consistently demonstrates superior periodontal and esthetic outcomes compared with conventional horizontal preparations.

## 4. Discussion

The design of tooth preparation is a decisive factor in preserving periodontal health and ensuring the long-term success of fixed prosthodontic restorations. The location and geometry of the finishing line directly influence marginal adaptation, plaque retention, and gingival response.

Evidence shows that CAD/CAM-fabricated zirconia restorations provide superior marginal fit and reduced gingival inflammation compared with conventionally fabricated metal–ceramic prostheses [27]. The smoother surfaces and more precise emergence profiles achieved through digital workflows contribute to plaque control and enhance periodontal stability. These findings highlight the importance of both material selection and fabrication technology in reducing biological complications.

Importantly, digital workflows are not only a matter of technical precision but have direct biological implications. By enabling accurate transfer of the provisional emergence profile, intraoral scanning and CAD/CAM systems facilitate a harmonious prosthesis–gingiva interface [28]. This translates into lower gingival inflammation, more stable probing depths, and reduced risk of marginal recession compared with conventional workflows. The vertical position of restoration margins is equally critical. Studies have consistently demonstrated that subgingival margins are associated with increased inflammation due to plaque retention and marginal misfit, whereas supragingival margins are more favorable for periodontal health and facilitate maintenance [9]. Preservation of the biologic width and respect for the supracrestal tissue attachment remain essential principles in restorative dentistry.

Long-term studies confirm that BOPT maintains stable soft tissue conditions with minimal recession and high patient satisfaction [4,19,20,29]. By contrast, conventional preparations such as chamfer and shoulder, although predictable and widely adopted, have been linked to progressive gingival recession and loss of papillary height over time, particularly in the anterior esthetic zone [7,8]. Recent in vitro data further suggest that horizontal preparations require greater tooth reduction in anterior teeth compared with BOPT, while BOPT achieves comparable retention with more conservative tissue removal [3]. A 10-year prospective trial also confirmed that restorations performed with BOPT maintain superior soft tissue stability compared to horizontal designs, reinforcing its clinical relevance [19].

Clinically, preparation design should be individualized: BOPT offers advantages for thin gingival biotypes and anterior esthetic cases, while chamfer and shoulder remain reliable in posterior regions with lower esthetic demands [4,8]. In high-risk patients, supragingival margins and minimally invasive designs are preferable [7,8,9,30]. The integration of digital workflows is also pivotal, as intraoral scanning and CAD/CAM technologies increase accuracy and reproducibility, particularly in BOPT cases where the exact transfer of the provisional emergence profile is crucial [21,22]. Beyond technical precision, this transferability directly impacts clinical outcomes: accurate reproduction of provisional contours contributes to lower gingival inflammation, maintenance of stable probing depths, and improved long-term margin stability [31]. Digital workflows therefore play not only a technical but also a biological role in supporting periodontal health.

Most available studies are prospective cohorts or case series with limited sample sizes, and heterogeneity in design and outcomes makes direct comparison difficult. This underscores the need for well-designed randomized controlled trials with long-term follow-up. In summary, while chamfer and shoulder preparations remain reliable and widely practiced, BOPT offers distinctive biological and esthetic benefits, especially in high-risk and esthetically demanding situations. The available evidence suggests that BOPT may offer biological and esthetic advantages in selected cases, but the heterogeneity and limited scale of current studies prevent definitive conclusions. Widespread adoption should await further clinician training supported by robust randomized trials and long-term data [4,7,8,19,20].

Additional contributions support the relevance of preparation design: Hategan et al. linked finish line geometry with periodontal health [32], while Petrescu et al. emphasized the biological determinants of esthetic outcomes in non-metallic prostheses [33]. Novel diagnostic methods such as optical coherence tomography [34] and micro-CT [35] may refine the evaluation of restorative interfaces. Furthermore, advances in smart biomaterials, including magnetorheological elastomers [36], illustrate potential future directions, although their direct application to tooth preparation design remains exploratory.

Recent advances in CAD/CAM technologies, both subtractive and additive, have enhanced the clinical relevance of preparation design by enabling precise finishing line reproduction, virtual gingiva modeling, and accurate transfer of provisional contours to definitive restorations [10,19,20]. These digital workflows contribute to improved marginal adaptation and emergence profile control, thus supporting periodontal stability and esthetic integration [11,21]. Nevertheless, long-term outcomes remain closely dependent on respecting the supracrestal tissue attachment and ensuring patient-related factors such as post-restorative care and adequate oral hygiene [7,8,9]. This is particularly relevant in patients with advanced periodontal disease, where restorative strategies must be carefully balanced with periodontal stability to achieve long-term success [37,38,39,40,41].

### Limitations and Clinical Implications

Most of the available evidence regarding tooth preparation design is derived from prospective cohort studies and case series, often with relatively small sample sizes and heterogeneous methodologies. A 10-year prospective study confirmed the long-term stability of BOPT restorations [19], but randomized controlled trials are still lacking. Previous findings have also shown that subgingival margins increase inflammatory risk compared to supragingival designs [9], highlighting the importance of respecting biological width and tissue health. Furthermore, recent investigations demonstrate that margin design and restorative material directly influence mechanical integrity and periodontal outcomes, emphasizing the need for well-structured comparative trials [27].

Clinically, preparation design should always be individualized according to the patient’s gingival biotype, esthetic requirements, and periodontal risk profile, ensuring that restorative strategies are tailored to both biological principles and patient-specific needs [8,19].

The reliability of conclusions is limited by the absence of a formal risk of bias assessment, as well as by heterogeneity in the included studies regarding design, follow-up periods, and clinical outcomes. These factors should be taken into account when interpreting the findings.

## 5. Conclusions

The preservation of periodontal health and stability is closely dependent on tooth preparation design. Respecting the biologic width, maintaining supragingival margins whenever possible, and ensuring a harmonious interaction between the restoration and soft tissues remain fundamental principles for long-term clinical success [7,9]. Conventional horizontal designs such as chamfer and shoulder remain predictable and widely used, but may predispose to inflammation and recession, especially in esthetic areas [8].

The Biologically Oriented Preparation Technique (BOPT) represents an alternative concept by promoting gingival adaptation to the restoration rather than obliging the prosthesis to conform to pre-existing contours. Long-term evidence demonstrates gingival thickening, stable probing depths, and improved margin stability with BOPT [4,19,20].

Nevertheless, the choice of preparation design should be individualized according to gingival biotype, esthetic requirements, and periodontal risk profile, to optimize both biological and functional outcomes. Clinically, preparation design should be individualized based on gingival biotype, esthetic requirements, and periodontal risk profile, balancing biological preservation with functional demands.

In summary, while BOPT demonstrates encouraging results regarding gingival stability and esthetic integration, its advantages over conventional preparations remain to be validated in large, well-designed clinical trials. Until such evidence is available, BOPT should be considered a promising but not yet proven alternative within the prosthodontic armamentarium.

## Figures and Tables

**Table 1:** Comparative clinical outcomes of Biologically Oriented Preparation Technique (BOPT) versus conventional horizontal preparations (Chamfer and Shoulder).

Parameter	BOPT (Vertical)	Chamfer (Horizontal)	Shoulder (Horizontal)
Gingival thickness	Mean increase of 0.3–0.5 mm over 6 years; stable mucosal volume at long-term follow-up [4,17,19,20]	Minimal change; may thin over time [3,8]	Minimal change; possible reduction in thin biotypes [3,8]
Gingival margin stability	High stability; mean recession <0.2 mm after 6 years; very few cases of recession reported at 6–10 years [4,19,20]	Recession commonly 0.3–0.6 mm at 5 years, especially in esthetic areas [7,8]	Recession often >0.5 mm in long-term studies, more pronounced than chamfer [8]
Probing depth	Stable probing depths; mean variation <0.2 mm at 10 years [17,19]	Initially low, but may increase by 0.3–0.5 mm after 2–3 years [8,9]	Similar to chamfer; possible increase with subgingival margins [8,9]
Gingival inflammation/BOP	Low inflammation; plaque index decreased over 6 years; at ~4 years, ≈12% of teeth showed BOP [17,18,19,20]	Plaque index often >25%; BOP >25% at 2–3 years with subgingival margins [8,23]	BOP frequently >30% when margins placed subgingivally [8]
Tooth structure reduction	0.6–0.8 mm in anterior teeth (in vitro) [3]	1.0–1.2 mm in anterior teeth (in vitro) [3]	1.2–1.5 mm in anterior teeth (in vitro); least conservative [3]
Esthetic outcomes	>90–95% patient satisfaction at 6–10 years [17,18,19,20]	~70–80% at 5 years; tends to decrease over time [8,23]	Acceptable initially, but less flexible for contouring; ~70% satisfaction [8]
Survival rates of restorations	>95% at 6 years; 10-year data available but limited [17,18,19,20]	High survival short- to medium-term (~90% at 5 years), but more biological complications long-term [8]	Similar to chamfer; survival maintained ~90%, but with higher soft tissue complications [8]
Digital workflow applicability	Highly compatible with CAD/CAM and intraoral scanning; accurate transfer of emergence profile [21,22]	Conventional workflows well established; digital feasible but less critical [12]	Conventional workflows standard; less integration with digital emergence design

Comparative clinical outcomes of Biologically Oriented Preparation Technique (BOPT) versus conventional horizontal preparations (Chamfer and Shoulder). Data represent ranges reported in prospective clinical trials, systematic reviews, and in vitro studies [3,7,8,9,17,18,19,20]. BOP = bleeding on probing; CAD/CAM = computer-aided design/computer-aided manufacturing.

## Data Availability

Not applicable.

## Abbreviations

The following abbreviations are used in this manuscript:

BOPT	Biologically Oriented Preparation Technique
CAD/CAM	Computer-aided design/computer-aided manufacturing
